# Prediction of Potato Crop Yield Using Precision Agriculture Techniques

**DOI:** 10.1371/journal.pone.0162219

**Published:** 2016-09-09

**Authors:** Khalid A. Al-Gaadi, Abdalhaleem A. Hassaballa, ElKamil Tola, Ahmed G. Kayad, Rangaswamy Madugundu, Bander Alblewi, Fahad Assiri

**Affiliations:** 1 Precision Agriculture Research Chair (PARC), King Saud University, Riyadh, Saudi Arabia; 2 Department of Agricultural Engineering, College of Food and Agriculture Sciences, King Saud University, Riyadh, Saudi Arabia; 3 Saudi Agricultural Development Company (INMA), Wadi Eddawasir, Saudi Arabia; University of Vigo, SPAIN

## Abstract

Crop growth and yield monitoring over agricultural fields is an essential procedure for food security and agricultural economic return prediction. The advances in remote sensing have enhanced the process of monitoring the development of agricultural crops and estimating their yields. Therefore, remote sensing and GIS techniques were employed, in this study, to predict potato tuber crop yield on three 30 ha center pivot irrigated fields in an agricultural scheme located in the Eastern Region of Saudi Arabia. Landsat-8 and Sentinel-2 satellite images were acquired during the potato growth stages and two vegetation indices (the normalized difference vegetation index (NDVI) and the soil adjusted vegetation index (SAVI)) were generated from the images. Vegetation index maps were developed and classified into zones based on vegetation health statements, where the stratified random sampling points were accordingly initiated. Potato yield samples were collected 2–3 days prior to the harvest time and were correlated to the adjacent NDVI and SAVI, where yield prediction algorithms were developed and used to generate prediction yield maps. Results of the study revealed that the difference between predicted yield values and actual ones (prediction error) ranged between 7.9 and 13.5% for Landsat-8 images and between 3.8 and 10.2% for Sentinel-2 images. The relationship between actual and predicted yield values produced R^2^ values ranging between 0.39 and 0.65 for Landsat-8 images and between 0.47 and 0.65 for Sentinel-2 images. Results of this study revealed a considerable variation in field productivity across the three fields, where high-yield areas produced an average yield of above 40 t ha^-1^; while, the low-yield areas produced, on the average, less than 21 t ha^-1^. Identifying such great variation in field productivity will assist farmers and decision makers in managing their practices.

## Introduction

Achieving the maximum crop yield at the lowest investment is an ultimate goal of farmers in their quest towards an economically efficient agricultural production. Early detection of problems associated with crop yield can greatly help in reducing the loss and reaching the targeted yield and profit. Potato is classified as being the fourth major staple around the globe, which is still quickly attaining importance [1]. The growing interest in potato, along with the diminishing agricultural lands, introduces the need for germplasm yield enhancement, better crop protection and much more efficient and productive management systems. Prediction of potato crop yield prior to the harvest period can be very useful in pre-harvest and marketing decision making. Many studies [2, 3] showed that traditional methods of crop yield estimation could lead to poor crop yield assessment and inaccurate crop area appraisal. In addition, these methods normally depend on rigorous field data collection of crop and yield, which is a costly and time-consuming process.

Remote sensing (RS) and global positioning system (GPS) technologies can be used to assess the temporal variation in crop dynamics, including crop yield and its spatial variability [4]. Visible (blue, green and red) and near infrared (NIR) portions of the electromagnetic spectrum have already proven their effectiveness in accessing information on crop type, crop health, soil moisture, nitrogen stress and crop yield [5–13]. Advancement in RS techniques enhanced the use of multispectral images as an effective tool in determining and monitoring vegetation conditions, crop stress and crop yield prediction. Liu and Kogan [14] revealed that remote sensing data offered exceptional spatial and temporal land surface characteristics, including the environmental impacts on crop growth. Numerous studies have reported that there could be a good correlation between the vegetation indices provided by the RS techniques and the crop yield and biomass [14–17]. A crop yield research that is conducted at a regional scale, which employs coarse or low-resolution satellite images, can provide a broader information on the crop canopy conditions and crop yield estimates. Hence, decisions in the quantitative export and import of the product within the region could be made in assured way.

Prediction of crop yields is typically associated with certain agronomic variables (density, vigor, maturity and disease), which can be used as yield indicators. Remote sensing offers a close diagnosis of plant health; however, the spectral reflectance of the crop is dependent on phenology, stage type and crop health. Several studies [4, 6, 18–22] have focused on crop growth analysis using normalized difference vegetation index (NDVI) to enhance precision agriculture. Research in plant life monitoring has proven that NDVI is associated with the leaf area index (LAI) and the photosynthetic activity of crops. The NDVI is an indirect way of measuring the primary productivity through its quasi-straight line relation using the Fraction of Absorbed Photosynthetically Active Radiation (FAPAR) [23] and [24]. Also, Baez-Gonzalez [6] used Landsat ETM (enhanced thematic mapper) data with an NDVI model to estimate corn yield, where a prediction error of 9.2% in the yield was determined. Yang [25] used the United States Department of Agriculture (USDA) EPIC model to predict yield, where the difference between recorded and predicted yield was less than 10%. Baez-Gonzalez [18] modeled a corn yield with NDVI generated from NOAA Advanced High-Resolution Radiometer (AVHRR) images. A study by Gopalapillai and Tian [19] reported correlation coefficient (r) values varying from 0.13 to 0.98 for predicting corn yield from nine different fields using a span of two-year datasets. They used aerial pictures of the corn plots and calculated NDVI to predict yield, where the average correlation coefficient (r) between the NDVI and the yield over all the nine fields was determined at 0.54. On the other hand, soil adjusted vegetation index (SAVI) was used in some studies as it showed a tendency to minimize soil brightness, a phenomenon which has been addressed by Miura [26] and Lamb [27]. Jayanthi [28] carried out research on yield estimation of potato integrating the SAVI from high resolution airborne multispectral imagery and developed various yield models. Huete [29] introduced a soil calibration element in the NDVI equation to take into account the very first order optical interactions between soil and vegetation. Bala and Islam [30] used TERRA MODIS images to estimate a potato yield, where a prediction error of 15% was determined using ground truth data collected from 50 fields.

In addition to providing a decision support tool and revenue expectation, the predicted yield maps can be used as spatial databases for the implementation of variable rate technology (VRT) systems to achieve a precise application of field-level inputs in order to optimize production across the entire field. Therefore, this study was designed to provide a means of early prediction of potato yield (i.e. prior to the harvest period) using multispectral satellite remote sensing on a field scale. However, the specific objectives of this study were (i) to obtain an empirical equation for the early prediction of potato yield using multispectral images in conjunction with field collected potato yields, (ii) to determine the suitable growth stage for early prediction of potato yield, and (iii) to classify the obtained yield maps into distinct zones for the implementation of precision agriculture activities.

## Materials and Methods

### Study area

**Fig 1:**
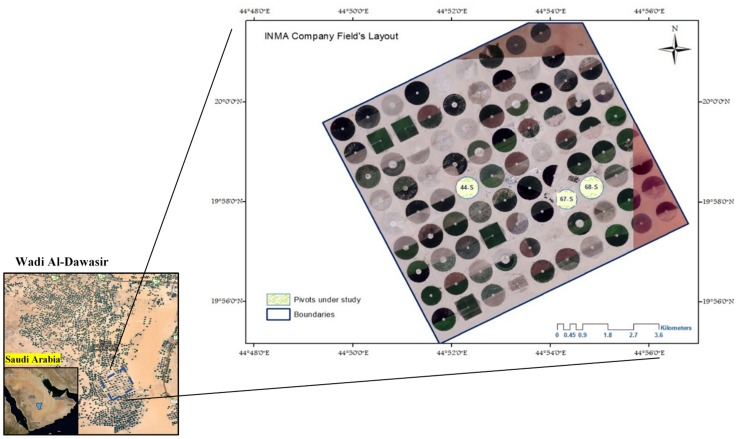
Location of the study fields.

This study was conducted on three 30 ha center pivot irrigated agricultural fields of Saudi Agricultural Development Company (INMA) in Wadi Al-Dawasir area south of Riyadh, the capital city of Saudi Arabia. The study area was located within the latitudes of 19.90° and 20.33° N and the Longitudes of 44.81° and 44.95° E (Fig 1).

Wadi Al-Dawasir region is one of the major irrigation water abstractions from Al-Wajid Aquifer. Agriculture in this region is dominated by developed agriculture enterprises that operate modern pivot irrigation systems. The size of the center pivot fields varies from 30 to 60 ha, where one farm can contain hundreds of fields irrigated with a number of aquifer wells. The main crops grown in winter are wheat, potato, tomato and melon. Fodder crops, including the biennial multi-cut crops of alfalfa and Rhodes grass, are grown throughout the year; however, inactive during winter. Meteorological features of the region are speckled. Diurnal temperature varies from 6°C (winter) to 43°C (hot summers) with an annual mean temperature of 27.4°C. The mean annual rainfall is around 37.6 mm [31].

### Satellite Images

Cloud-free satellite images of landsat-8 and sentinel-2 (Table 1) for the study period (January 26^th^, 2016 to March 14^th^, 2016) were downloaded from the archives of USGS Earth Explorer website (http://earthexplorer.usgs.gov/). Landsat-8 (the Operational Land Imager (OLI)) was calibrated using the data-specific utilities of ENVI (Ver. 5.3) software program, in which the sensor digital numbers were converted into spectral radiance in order to measure the amount of electromagnetic radiation reflected from a spot on the surface. The spectral radiance (Lλ) was determined using the calibration coefficients from the image metadata. Subsequently, reflectance images were generated from the obtained radiance. Various atmospheric correction techniques (dark object removal, haze removal, cloud masking, etc.) were applied to correct the sensor radiance for atmospheric effects by mathematically modeling the physical inclinations of the radiation as it passes through the atmosphere. Image enhancement and histogram stretch (linear) were also carried out. Sentinel-2, in turn, is based on a satellite constellation deployed in polar sun-synchronous orbit. While ensuring data continuity of previous Spot and Landsat multi-spectral missions, Sentinel-2 can even offer broad improvements, such as a unique blend of global coverage with a wide field of view (290 km), a very high revisit (5 days with two satellites), a high resolution (10 m, 20 m and 60 m) and multi-spectral imagery (13 spectral bands in visible and shortwave infra-red domains). Image provided for this study was level- 1C, which was an ortho-rectified top of atmosphere reflectance with a sub-pixel multi-spectral and multi-date registration; a cloud and land/water mask was associated to the product. The Level-1C processing is enhanced by algorithms of resampling and cloud and land/water masks computations, which make it a stage that lead to the computation of level-1B (Top of Atmosphere (TOA) reflectance) product. The spectral bands span from the visible and the near infrared to the short wave infrared. Four bands at 10 m ground resolution include the classical blue (490 nm), green (560 nm), red (665 nm) and near infrared (842 nm) bands specialized in land applications.

**Table 1:** Used satellite images.

Sl. No	Sensor	Dates of Pass	Path/Row/Tile No.	Spatial resolution
1	Landsat-8 (OLI)	12 January, 26 January, 11 February, 27 February and 10 March 2016	166 / 46	30 (m)
2	Sentinel-2	11 February 2016	T38QMH (Orbit No. 49)	10 (m)

Vegetation indices (VIs), such as NDVI and SAVI, were extracted from the pre-processed images and used for yield prediction. In addition, the cumulative value of the generated indices (CNDVI and CSAVI) was also examined for the development of potato yield prediction algorithms. The NDVI and SAVI were calculated using red (ρ_RED_) and near-infrared (ρ_NIR_) spectral bands of Landsat-8 and Sentinel-2 as in Eqs 1 and 2 [32] and [33].

$$\text{NDVI}=\frac{\left(\rho_{NIR}-\;\rho_{RED}\right)}{\left(\rho_{NIR}+\;\rho_{RED}\right)}$$ (1)

$$\text{SAVI}=\frac{\left(\rho_{NIR}-\;\rho_{RED}\right)}{\left(\rho_{NIR}+\;\rho_{RED}+\text{L}\right)}\;(1+L)$$ (2)

Where, L is the canopy background adjustment factor. An L value of 0.5 in reflectance space was found to minimize soil brightness variations and eliminate the need for additional calibration for different soils [28].

**Fig 2:**
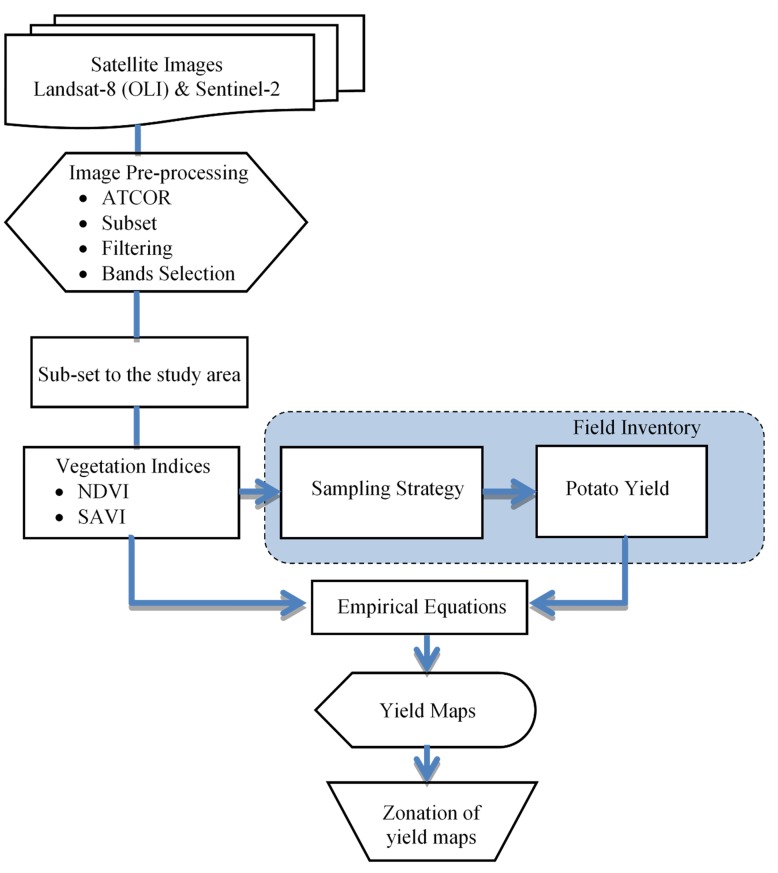
Yield prediction map generation and analysis.

As depicted in Fig 2, the obtained satellite images were analyzed for vegetation indices and used for the determination of sample locations for in-situ potato yield collection. The field collected potato yield data points were correlated to the corresponding VIs in order to generate algorithms for the early prediction of potato yield for the given fields.

### Sampling strategy for in-situ potato yield collection

Three center-pivot irrigated fields (67-S, 68-S and 44-S) were considered as sample fields for this study. In each of the three study fields, stratified random sampling-based sixty locations (60 points: 45 for model generation and 15 for validation) were determined for in-situ potato (tuber) yield data collection. These locations were distributed across each of the experimental fields using the randomizer function of ENVI (Ver. 5.3) software program according to a prescription map generated based on vegetation cover variability and were located in the field with the help of a GPS receiver (Trimble GeoXH). The cumulative NDVI (CNDVI) layer, which is a compilation of the four extracted NDVI images during the growth stages, was used as a source for the stratification base. The stratified random sampling provided by ENVI software, which also called proportional or quota random sampling, involves splitting up the population (the complete classification image) into homogeneous subgroups (the individual classes). After that, a basic random sample in every subgroup was selected. The proportionate sampling type, which was used in this study, tends to create sample sizes that are proportional to the size of the classes (the greater the class, the more samples are to be drawn from it).

Intensive fieldwork from April 10^th^ to April 16^th^, 2016 was carried out 2 to 3 days prior to the harvest time of each field to assure the steadiness of crop status. In-situ collection of potato yield (actual yield) was achieved by harvesting potato over a 3 m^2^ area at each sampling point. The harvested potatoes were weighed and up-scaled to the common yield unit (t ha^-1^). The composite samples selected from the aggregates were analyzed for the determination of dry matter (DM) using the hydrometry test. Out of the collected 60 samples, 75% were used for model generation and the rest (25%) were considered for model validation.

### Prediction of potato yield

In order to reveal the relationship between actual yield and VIs and to generate an empirical equation for the prediction of potato yield, a scatter plot was applied between the actual yield and the RS based generated VIs (NDVI, SAVI, CNDVI and CSAVI) across the growth period. The yield samples (45 yield points) availed for model construction were analyzed for Pearson correlation (linear) coefficient against the single-date VI (NDVI and SAVI) and the multi-date cumulative VIs (CNDVI and CSAVI). Moreover, growth stage-wise correlation coefficients were analyzed for the best fit empirical equations; hence, the most suitable VI for the prediction was identified accordingly. The optimum growth stage, at which the VIs were highly correlated to the yield, was assessed for the determination of the appropriate time of potato yield prediction prior to the harvest period.

### Model validation and accuracy assessment

The empirical models used for the prediction of potato yield were validated against the in-situ potato yields (actual yields). For this purpose, a scatter plot was applied between the 25% of the actual yield (apart from the samples used for model generation) and the predicted yields, and then analyzed for Pearson correlation coefficient (R^2^). Performance indicators, such as the root mean square error (RMSE) and the mean bias error (MBE) were determined in addition to the Nash-Sutcliffe Efficiency (NSE), which is a normalized statistic that establishes the relative value of the residual variance in comparison to the measured data variance [34].

### Zonation of predicted yield maps

In the view of implementing precision agriculture practices and to provide an insight of the field productivity variation for better future management, the predicted yield maps were classified into five distinct zones based on their productivity. These zones included very high (above 40 t ha^-1^), high (35–40 t ha^-1^), medium (30–35 t ha^-1^), low (20–30 t ha^-1^) and very low (below 20 t ha^-1^) productivity zone. The pixel value, at each location in the image, represented the average yield (t ha^-1^) within the pixel dimensions. Pixels with equal or close yield values were compiled to assess the zonal yield.

## Results and Discussion

### In-situ potato yield

The potato yield samples collected at the three fields from the pre-allocated points were statistically analyzed to assess the spatial variability of the yield and the productivity of different zones within one field. Table 2 presents some statistics of the collected potato samples.

**Table 2:** Descriptive statistics of the actual potato yield.

Field Number		67-S	68-S	44-S
**Number of Samples**		45	45	45
**Actual Yield (t ha**^**-1**^**)**	Minimum	4.7	18.9	27.6
	Maximum	48.5	45.3	62.7
	Mean	34.1	36.7	42.5
	Std. Deviation	12.3	5.6	7.0
	Std. Error	1.8	0.8	1.1

### Potato yield prediction models

Linear regression analysis showed that the relationships between the actual crop yield and VIs were varying throughout the growth period (Table 3). Results revealed that the early and late stages of crop life showed the least correlations. This can be attributed to that at the early crop stages, reflection from vegetation cover is highly noised by the soil surface. On the other hand, at late stages (i.e. when the tubers are completely riep), potato leaves tend to turn into yellow (i.e. reduced chlorophyll) [35]. However, the best-fit models were obtained with NDVI, SAVI, CSAVI and CNDVI. The highest correlation (R^2^) value was observed to be found 60 to 70 days after planting dates of the potato crop (Table 3). For Landat-8 data, the best-fit models were obtained with CSAVI, NDVI and SAVI for fields 67-S, 68-S and 44-S, respectively. However, for sentinel-2, SAVI provided the best-fit for all of the studied three fields.

**Table 3:** Potato growth stage-wise VIs and their relationship with the actual potato yield.

Pivot No.		67-S			68-S			44-S		
Sowing Date		12 December 2015			15 December 2015			18 December 2015		
Sensor			Landsat-8 (OLI)	Sentinel-2		Landsat-8 (OLI)	Sentinel-2		Landsat-8 (OLI)	Sentinel-2
*Image Date*	*Vegetation Index*	*Crop Age (days)*	*Equation (R*^*2*^*)*	*Equation (R*^*2*^*)*	*Crop Age (days)*	*Equation (R*^*2*^*)*	*Equation (R*^*2*^*)*	*Crop Age (days)*	*Equation (R*^*2*^*)*	*Equation (R*^*2*^*)*
**26 January 2016**	*NDVI*	45	0.48		42	0.12		39	0.18	
	*SAVI*		0.47			0.14			0.14	
**11 February 2016**	*NDVI*	61	0.50	0.49	58	0.13	0.23	55	0.30	0.42
	*SAVI*		0.50	0.50		0.14	0.22		0.28	0.42
**27 February 2016**	*NDVI*	77	0.48		74	0.06		71	0.22	
	*SAVI*		0.49			0.06			0.21	
**14 March 2016**	*NDVI*	92	0.34		89	0.01		86	0.37	
	*SAVI*		0.29			0.02			0.31	
	*CNDVI*		0.50			0.11			0.40	
	*CSAVI*		0.50			0.12			0.39	

Table 4 presents the obtained prediction equations that have been used to generate potato yield maps. Prediction algorithms from Landsat-8 showed a preference of using different VIs for yield prediction (CNDVI, NDVI and SAVI for fields 67-S, 68-S and 44-S, respectively). This can be attributed to the effect of the relatively coarse (30 m) spatial resolution which hinders the optimum representation of the vegetation cover reflection. In addition, the variation in crop age over the three fields (difference in cultivation dates) could be a cause of different indices selection. However, the yield was found to be correlating well with the SAVI (as in Sentinel-2) because SAVI minimized the soil background reflection effects caused by natural variability in surface reflection [29].

**Table 4:** The best fit equations used for the prediction of potato yield in the three fields.

Pivot No.	Sentinel-2	Pearson R^2^	Landsat-8	Pearson R^2^
**67 –S**	Yield(t ha^−1^) = 60.012 × SAVI + 6.5005	0.65	Yield(t/ha) = 30.592 × CSAVI + 12.575	0.65
**68 –S**	Yield(t ha^−1^) = 41.347 × SAVI + 16.621	0.45	Yield(t/ha) = 30.071 × NDVI + 13.175	0.49
**44—S**	Yield(t ha^−1^) = 110.84 × SAVI + 15.845	0.47	Yield(t/ha) = 119.79 × SAVI + 11.779	0.39

What can be revealed from the equations is that each prediction equation must be handled as sensor and location specific. Hence, for each field there are two prediction equations from two different satellite images. In general, the mid-growth stage (60–70 days after planting) was found to be the best time for yield prediction for potato crop.

### Accuracy of models used in potato yield prediction

**Fig 3:**
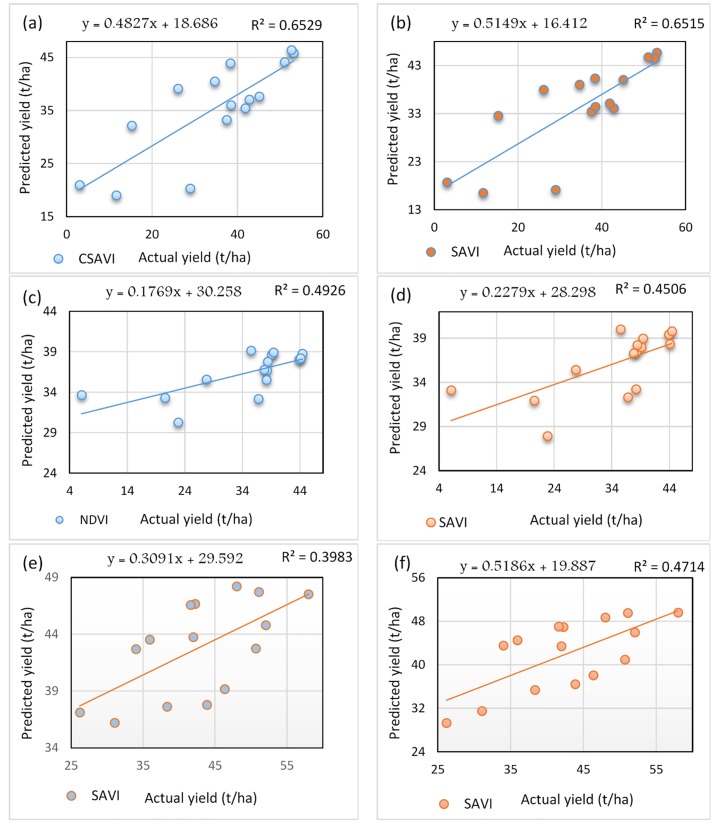
Correlation between actual and predicted yield for pivot 67-S with (a) CSAVI from Landsat-8 and (b) SAVI from Sentinel-2, pivot 68-S with (c) NDVI from Landsat-8 and (d) SAVI from Sentinel-2 and pivot 44-S with (e) SAVI from Landsat-8 and (f) SAVI from Sentinel-2.

Accuracy of the obtained empirical (yield prediction) models was assessed against the actual yield. The highest correlation was observed for the field number 67-S, where both cumulative SAVI (Landsat-8) and single-date SAVI (Sentinel-2) resulted in a similar R^2^ value of 0.65. Validation results for field 68-S showed R^2^ values of 0.49 and 0.45, when potato yield was correlated with NDVI (Landsat-8) and SAVI (Sentinel-2), respectively. Furthermore, for field 44-S, the R^2^ values of 0.39 and 0.47 were obtained by incorporating SAVI from Landsat-8 and Sentinel-2, respectively (Fig 3).

Results of the performance indicators used in the validation of prediction model are provided in Table 5. The correlation between the yield predicted by the developed models and the actual yield revealed consistent similarity in terms of yield spatial and quantitative distribution with high significances.

**Table 5:** Model validation and performance indicators.

Field No.	Sentinel-2						Landsat-8					
	Pearson R^2^	Std. Dev.	RMSE (%)	Sig. (1-tailed)	MBE (%)	NSE	Pearson R^2^	Std. Dev.	RMSE (%)	Sig. (1-tailed)	MBE(%)	NSE
**67—S**	0.65	0.15	8.80	0.000	-2.00	0.58	0.65	0.286	8.74	0.000	1.30	0.62
**68—S**	0.45	0.063	4.96	0.001	-6.00	0.25	0.49	0.05	5.25	0.008	-5.40	0.31
**44—S**	0.47	0.041	5.36	0.000	-0. 11	0.38	0.39	0.03	5.97	0.000	1.60	0.46

Pearson R^2^: Coefficient of determination

Std. Dev.: The standard deviation

RMSE (%): The root-mean-square error

Sig. (1-tailed): A Statistical significance

MBE (%): The mean bias error

NSE: The Nash-Sutcliffe Efficiency

The obtained validation accuracies were in agreement with previous findings, where the prediction was conducted on different crops using different space borne sensors [33] and [36]. As stated by [28], yield prediction accuracy increased with the increase of the density of observation points at the field. Given that statement, it was noticed from the accuracy investigation that high values of R^2^ could be obtained at a field when zonal-management strategy was applied in terms of sufficient observation points within each field zone. Temporal variability also affects the validation accuracy, where the results of applying the obtained model for the subsequent growth periods would determine the applicability of the tested model for a continuous prediction of potato yield.

Table 6 represents a comparison between the actual yields and the predicted potato yields of the entire fields (67-S, 68-S and 44-S) after applying the prediction equations to the whole field’s pixels (not only the 45 locations). Thus, the prediction errors were described as results of comparing the total actual collected yields versus the total predicted ones. Total field’s yield data were acquired after performing the full harvest of the three fields. From Table 6, it can be seen that the analyzed image from Sentinel-2 produced a better prediction accuracy for fields 67-S and 68-S (prediction errors of 3.8% and 7.5%, respectively) compared to that produced by Landsat-8 images (prediction errors of 7.9% and 13.5%, respectively). This was attributed to the high spatial resolution of Sentinel-2 images, which enabled the reduction of the generalization in crop spectral reflection and delineation of sharp field boundaries, so that noise from the neighboring soils was removed. However, the prediction errors from Landsat-8 (9.1%) and Sentinel-2 (10.2%) in field 44-S were very much indifferent.

**Table 6:** Total predicted vs. actual yields.

**Landsat—8**						
	**Field: 67-S**		**Field: 68-S**		**Field: 44-S**	
	**Fresh weight (Ton)**	**Dry Matter (%)**	**Fresh Weight (Ton)**	**Dry Matter (%)**	**Fresh Weight (Ton)**	**Dry Matter (%)**
Predicted yield*	1040	20.8	1060	25.0	1123	20.4
**Actual yield** *	957.26	20.8–21.0	1225.48	25.0	1235.69	20.4
Prediction Error * (%)	***7*.*9***		***13*.*5***		***9*.*1***	
**Sentinel- 2**						
	**Field: 67-S**		**Field: 68-S**		**Field: 44-S**	
	**Fresh Weight (Ton)**	**Dry Matter (%)**	**Fresh Weight (Ton)**	**Dry Matter (%)**	**Fresh Weight (Ton)**	**Dry Matter (%)**
Predicted yield*	995.00	20.8	1133.67	25.0	1110	20.4
**Actual yield** *	957.26	20.8–21.0	1225.48	25.0	1235.69	20.4
Prediction Error * (%)	***3*.*8***		***7*.*5***		***10*.*2***	

* Predicted yield, actual yield and prediction error were determined based on fresh potato weight.

### Zonal analysis of the classified yield maps

**Fig 4:**
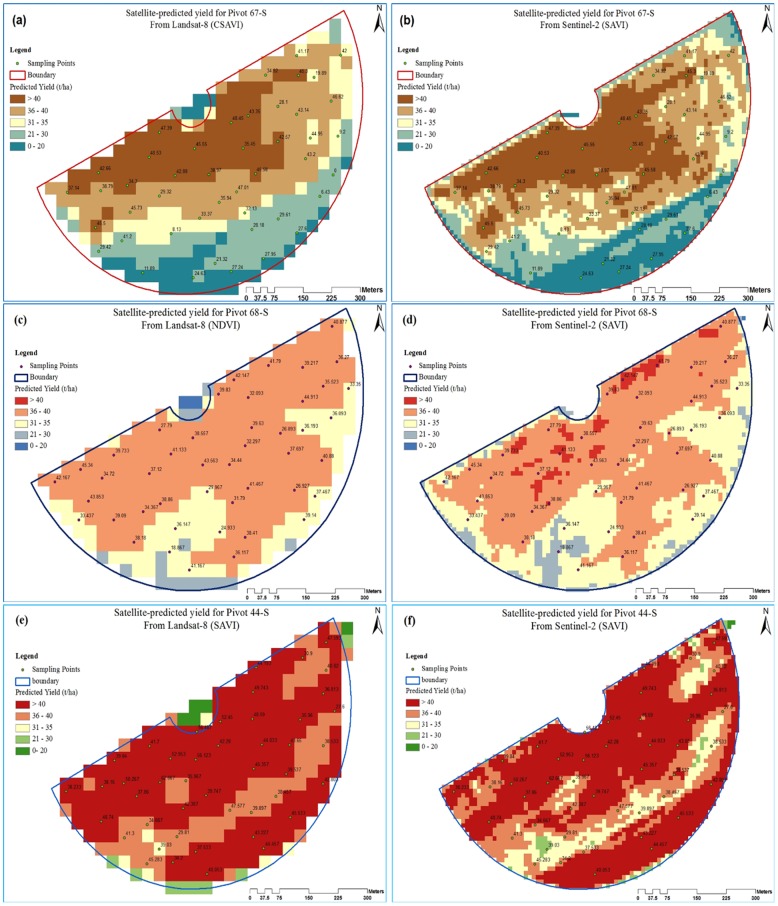
Maps of predicted potato yield for (a) pivot 67-S using CSAVI from Landsat-8, (b) pivot 67-S using SAVI from Sentinel-2, (c) pivot 68-S using NDVI from Landsat-8, (d) pivot 68-S using SAVI from Sentinel-2, (e) pivot 44-S using SAVI from Landsat-8 and (f) pivot 44-S using SAVI from Sentinel-2.

The predicted yield maps of the three study fields (Fig 4) were developed from the Landsat-8 and Sentinel-2 satellite images employing the different indices shown in Table 4 and Fig 3. These maps showed a considerable variation in yields within each field as represented by the five classes. For all the study fields, it can be observed that the very-low-yield class was mainly distributed across the field boundaries. This was attributed to the fact that pixels of this yield class were located at the cross-boundary between the vegetation and the bare soil areas (field’s end); where scarceness of the yield existed. On the other hand, the low VIs estimated values could probably be attributed to the reflections from the holistic surface covers (vegetation + bare soil), especially from the coarse resolution images of Landsat-8 compared to Sentinel-2. The yield classes of Landsat-8 and Sentinel-2 maps were described based on pixel count that associated with class creation. By comparing the yield potentials within the very-high-yield and the very-low-yield classes, a variation of yield ranging from 20 to 40 t ha^-1^ was found to exist within each field. The least yield values (0 to 20 t ha^-1^) were observed along the fields boundary, where the cumulative reflectance of soil and vegetation was detected at the field’s end.

**Fig 5:**
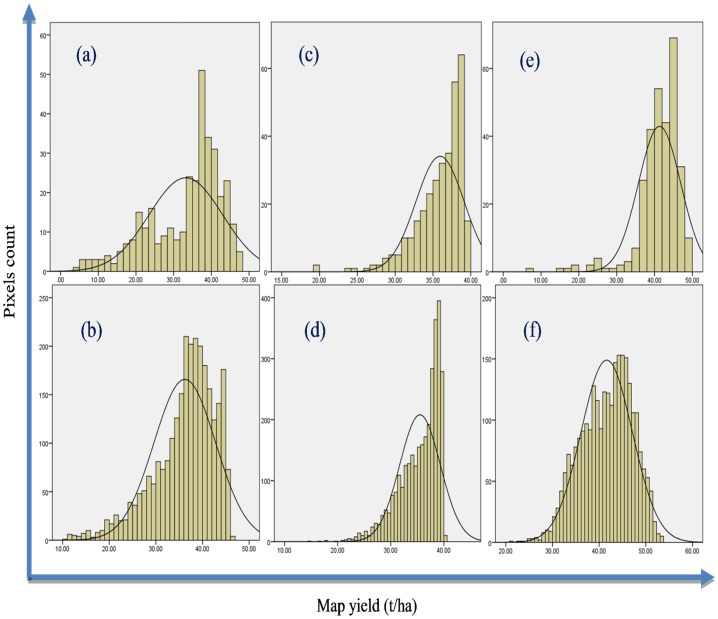
Histogram of potato yield for (a) pivot 67-S from Landsat-8, (b) pivot 67-S from Sentinel-2, (c) pivot 68-S from Landsat-8, (d) pivot 68-S from Sentinel-2, (e) pivot 44-S from Landsat-8 and (f) pivot 44-S from Sentinel-2.

The class-wise histograms of potato yield distributed across the studied fields were developed (Fig 5). From Fig 5, it can be seen that the most frequent productivity was achieved within the high-yield classes of fields 67-S and 68-S, with total absence of the very-high-yield class in field 68-S, which were observed to have average values of 37 t ha^-1^ for both fields and a yield range of 37 to 38 t ha^-1^ for field 44-S. On the other hand, the most productive areas (very-high-yield classes) were observed to be less frequent compared to the high-yield classes. These results highlighted the importance of improving the productivity over the three least yield classes (medium, low and very low) to increase the total field productivity through the use of potato yield monitoring techniques and a suitable management. On the other hand, it is believed that expanding the analysis to cover the environmental and soil influential factors can assist in the enhancement of the yield productivity. The normal distribution shown by graphs (e) and (f) revealed that the field 44-S produced a broad variety of yields within the five yield classes. Production with such variability could complicate the planning for the zone management in terms of following a specific type agricultural activity or applying fixed additive doses.

**Fig 6:**
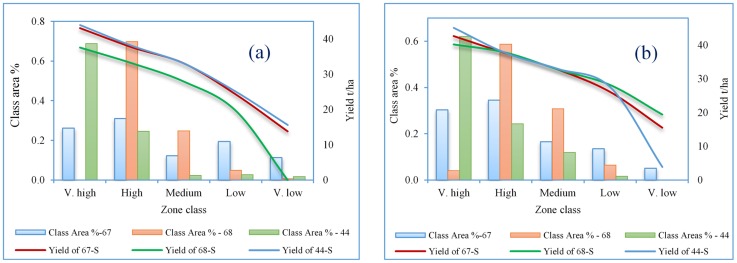
The variation in productivity classes of (a) Landsat-8 and (b) Sentinel-2 yield maps.

In order to demonstrate the importance of reclaiming the zones with the low-yields, a quantitative assessment of the predicted potato yield derived using both satellites (Landsat-8 and Sentinel-2) over the three fields was conducted. Fig 6 shows the relative variation in area versus yield classes for Landsat-8 and Sentinel-2 maps. The resulting analysis highlighted the potential of using precision agriculture in the enhancement of the productivity through managing the low yield locations for more profitability (vertical expansion in crop yield). From Landsat-8 yield maps, it was calculated that the area of very-high-yield class (yield ≥ 40 t ha^-1^) occupied 25% of the total area of field 67-S (Fig 6a); however, the same class occupied 68% of the total area of field 44-S. On the other hand, no such class (very-high-yield zone) was predicted for field 68-S, which indicated a lower field productivity that was limited to less than 40 t ha^-1^. Sentinel-2 maps, however, revealed that the very-high-yield classes (yield ≥ 40 t ha^-1^) occupied 30%, 4% and 62% of the total area for fields 67-S, 68-S and 44-S, respectively (Fig 6b). Prediction by Sentinel-2 of the very-high-yield class in field 68-S contradicted with the results from Landsat-8 for the same field. However, the actual yield observations for field 68-S agreed with the Sentinel-2 predictions. The contradiction could be attributed to the coarse spatial resolution of Landsat-8, in which the surface reflection was associated with multiple factors, such as soil, weeds, etc.

## Conclusions

A field study was conducted to provide an efficient and practical means of predicting potato tuber yield cultivated under a center pivot irrigation system using remote sensing techniques. The following conclusions are inferred from the study:

- The study provided an effective method to predict potato yield and map its spatial variability. Images from Landsat-8 (30 m resolution) and Sentinel-2 (10 m resolution) satellite vehicles were downloaded for the study area free of charge. The use of vegetation indices (VIs) extracted from the satellite images was found to provide an effective and an efficient means of potato yield prediction.
- Prediction of crops yield through analyzing their bioactivities using satellite images could avail considerable information to improve productivity, such as recognizing the variation in field productivity across the field. It has been noticed in this study that high-yield areas produced average yields of above 40 t ha^-1^; however, the low-yield areas produced, on the average, yields of less than 21 t ha^-1^. Displaying and highlighting such great variations in field productivity can help farmers and decision makers be aware of the problem; hence, adjust their management practices.

## Supporting Information

S1 Fig The ordinal process of yield collection (a) & (b) points allocation, (c) crop collection and (d) instantaneous weighing of collected crop. (TIF)

S1 File A descriptive video clip shows the data collection process at the experimental field. (WMV)

S1 Table Tables show the calculations of the prediction models and models validation. (XLSX)

S2 Table Statistical analyses of the collected yield. (XLS)

## References

[1] Haverkort AJ, Mac Kerron DKL. In: Potato Ecology and Modelling of Crops under Conditions Limiting Growth Proceedings. Eds. of the 2nd International Potato Modeling Conference 17–19 May 1995. vol. 3. Wageningen. 1995. (Series: Current Issues in Production Ecology, ISBN 978-0-7923rr3412-5), p. 396.

[2] Reynolds CA, Yitayew M, Slack DC, Hutchison CF, Huete AR, Petersen MS. Estimating crop yields and production by integrating the FAO Crop Specific Water Balance model with real-time satellite data and ground based ancillary data. International Journal of Remote Sensing, 2000, 21(18):3487–3508.

[3] Dadhwall VK, Ray SS. Crop assessment using remote sensing—Part II: Crop condition and yield assessment. Indian Journal of Agricultural Economics, 2000, 2 (1947)-4, 55–67.

[4] Taylor JC, Wood GA, Thomas G. Mapping yield potential with remote sensing. *Precis*. *Agric*. 1997 1, 713–720.

[5] Magri A. Soil test, aerial image and yield data as inputs for site-specific fertility and hybrid management under maize. *Precis*. *Agric*. 2005 6, 87–110.

[6] Baez-Gonzalez AD, Kiniry JR, Maas SJ, Tiscareno ML, Macias JC, Mendoza JL, et al Large-area maize yield forecasting using leaf area index based yield model. *Agron*. *J*. 2005 97, 418–425.

[7] Lobell DB, Ortiz-Monasterio JI, Asner GP, Naylor RL, Falcon WP. Combining field surveys, remote sensing, and regression trees to understand yield variations in an irrigated wheat landscape. *Agron*. *J*. 2005 97, 241–249.

[8] Doraiswamy PC, Moulin S, Cook PW, Stern A. Crop yield assessment from remote sensing. *Photogramm*. *Eng*. *Remote Sens*. 2003 69, 665–674.

[9] Tan G, Shibasaki R. Global estimation of crop productivity and the impacts of global warming by GIS and EPIC integration. *Ecol*. *Model*. 2003 168, 357–370.

[10] Lillesand TM, Keifer RW. *Remote Sensing and Image Interpretation*, 3^rd^ edition; John Willey and Sons: New York, NY, USA 1994 P. 750 *Remote Sensing* 2010, *2*

[11] Hassaballa AA, Althuwaynee OF, Pradhan B. Extraction of soil moisture from RADARSAT-1 and its role in the formation of the 6th December 2008 landslide at Bukit Antarabangsa, Kuala Lumpur. *Arabian Journal of Geosciences*. 2014 Vol. 7, no7, pp. 2831–2840.

[12] Hassaballa AA, Matori AB, Shafri HZM. Surface Moisture Content Retrieval from Visible/Thermal Infrared Images and Field Measurements. *Caspian Journal of Applied Sciences Research CJASR*. 2013 (2), pp. 182–189.

[13] Hassaballa AA, Matori AB. The Estimation of Air Temperature from NOAA/AVHRR Images and the study of NDVI-Ts impact, Case Study: The Application of Split-Window Algorithms over (Perak Tengah & Manjung) area, Malaysia. IEEE International Conference on Space Science and Communication (IconSpace), 12–13 July 2011. Page(s): 20–24.

[14] Liu WT, Kogan F. Monitoring Brazilian soybean production using NOAA/AVHRR based vegetation condition indices. *International Journal of Remote Sensing*. 2002 23(6):1161–1179.

[15] Gat N, Erives H, Fitzgerald GJ, Kaffka SR, Mass SJ. Estimating sugar beet yield using AVIRIS derived indices. http://makalu.jpl.nasa.gov/docs/ workshops/00_docs/gat_web.pdf. 2000.

[16] Groten SME. NDVI-crop monitoring and early yield assessment of Burkina Faso. Title REMOTE SENSING 14 1993 No. 8: 1495–1515.

[17] Rasmussen MS. Operational Yield forecast using AVHRR NDVI data: reduction of environmental and inter-annual variability. *International Journal of Remote Sensing*. 1997 18(5):1059–1077.

[18] Baez-Gonzalez AD, Chen P, Tiscareno-Lopez M, Srinivasan R. Using satellite and field data with crop growth modeling to monitor and estimate corn yield in Mexico. *Crop Science*. 2002 42, 1943–1949.

[19] GopalaPillai S, Tian L. In-field variability detection and yield prediction in corn using digital aerial imaging. *Trans*. *ASAE*. 1999 42, 1911–1920.

[20] Senay GB, Ward AD, Lyon JG, Fausey NR, Nokes SE. Manipulation of high spatial resolution aircraft remote sensing data for use in site specific farming. *Trans*. *ASAE*. 1998, 41, 489–495.

[21] Yang C, Anderson GL. Mapping grain sorghum yield variability using airborne digital videography. *Precis*. *Agric*. 2000 2, 7–23.

[22] Funk C, Budde M. Phenologically-tuned MODIS NDVI-based production anomaly estimates for Zimbabwe. *Remote Sens*. *Environ*. 2009 113, 115–125.

[23] Prince SD. High Temporal Frequency Remote Sensing of Primary Production Using NOAA AVHRR In *Applications of Remote Sensing in Agriculture*; Steven M.D., Clark J.A., Eds.; Butterworths: London, UK 1990 pp. 169–183.

[24] Los SO. Linkages between Global Vegetation and Climate: An Analysis based on NOAA Advanced Very High Resolution Data. Ph.D. Thesis, Vrije Universiteit, Amsterdam, the Netherlands. 1998.

[25] Yang P, Tan GX, Zha Y, Shibasak, R. Integrating remotely sensed data with an ecosystem model to estimate crop yield in north China. In Proceedings of XXth ISPRS Congress Proceedings Commission VII, WG VII/2, Istanbul, Turkey. 2004. pp. 150–156.

[26] Miura T, Huete AR, Yoshioka H. Evaluation of sensor calibration uncertainties on vegetation indices for MODIS. *IEEE Trans*. *Geosci*. *Remote Sens*. 2000 38, 1399–1409.

[27] Lamb DW, Weedon MM, Rew LJ. Evaluating the accuracy of mapping weeds in seedling crops using airborne digital imaging: Avena spp. in seedling triticale. *Weed Res*. 1999 39, 481–492.

[28] Jayanthi H. Airborne and Ground-Based Remote sensing for the estimation of Evapotranspiration and yield of Bean, Potato, and sugar beet crops. Ph.D dissertation. Utah State University, Logan, Utah 2003. 185 p.

[29] Huete AR. A soil-adjusted vegetation index (SAVI). *Remote Sensing of Environment*. 1988 vol. 25, issue 3, pp. 259–309.

[30] Bala SK, Islam AS. Correlation between potato yield and MODIS derived vegetation indices. *International Journal of Remote Sensing* 2009 30:10, 2491–2507.

[31] Elhag M. Evaluation of Different Soil Salinity Mapping Using Remote Sensing Techniques in Arid Ecosystems, Saudi Arabia. *Journal of Sensors*. Faso. *International Journal of Remote Sensing* 2016 14(8):1495–1515.

[32] Rouse J Jr, Haas RH, Schell JA, Deering DW. Monitoring vegetation systems in the Great Plains with ERTS. *NASA special publication* 1974 351, 309.

[33] Haig LAS. Crop yield estimation: Integrating RS, GIS, management and land factors. A case study of Birkoor and Kortigiri Mandals- Nizamabad district, India. Thesis submitted to the International Institute for Geo-information Science and Earth Observation in partial fulfillment of the requirements for the degree of Master of Science in Geoinformation Science and Earth Observation; Sustainable Agriculture 2003.

[34] Nash JE, Sutcliffe JV. River flow forecasting through conceptual models part I -A discussion of principles, Journal of Hydrology, 1979, 10 (3), 282–290

[35] Palaniswami MS, Peter KV. Tuber & Root Crops Horticulture Science Series, New India Publishing Agency 2008, Pitam Pura, New Delhi– 110 088, Vol. 09, Page 167.

[36] Sharma T, Sundha KS, Ravi N, Navalgund RR, Tomar KP, Chakravorty NVK, Das DK. Procedures for wheat yield prediction using Landsat MSS and IRS 1A data. *International Journal of Remote Sensing*. 1993 14:2509–2518.

