# Supplementary figures and images for: Prediction of Potato Crop Yield Using Precision Agriculture Techniques
Source: PLoS One. 2016 Sep 9;11(9):e0162219. doi: 10.1371/journal.pone.0162219 (PMC5017787; doi:10.1371/journal.pone.0162219)

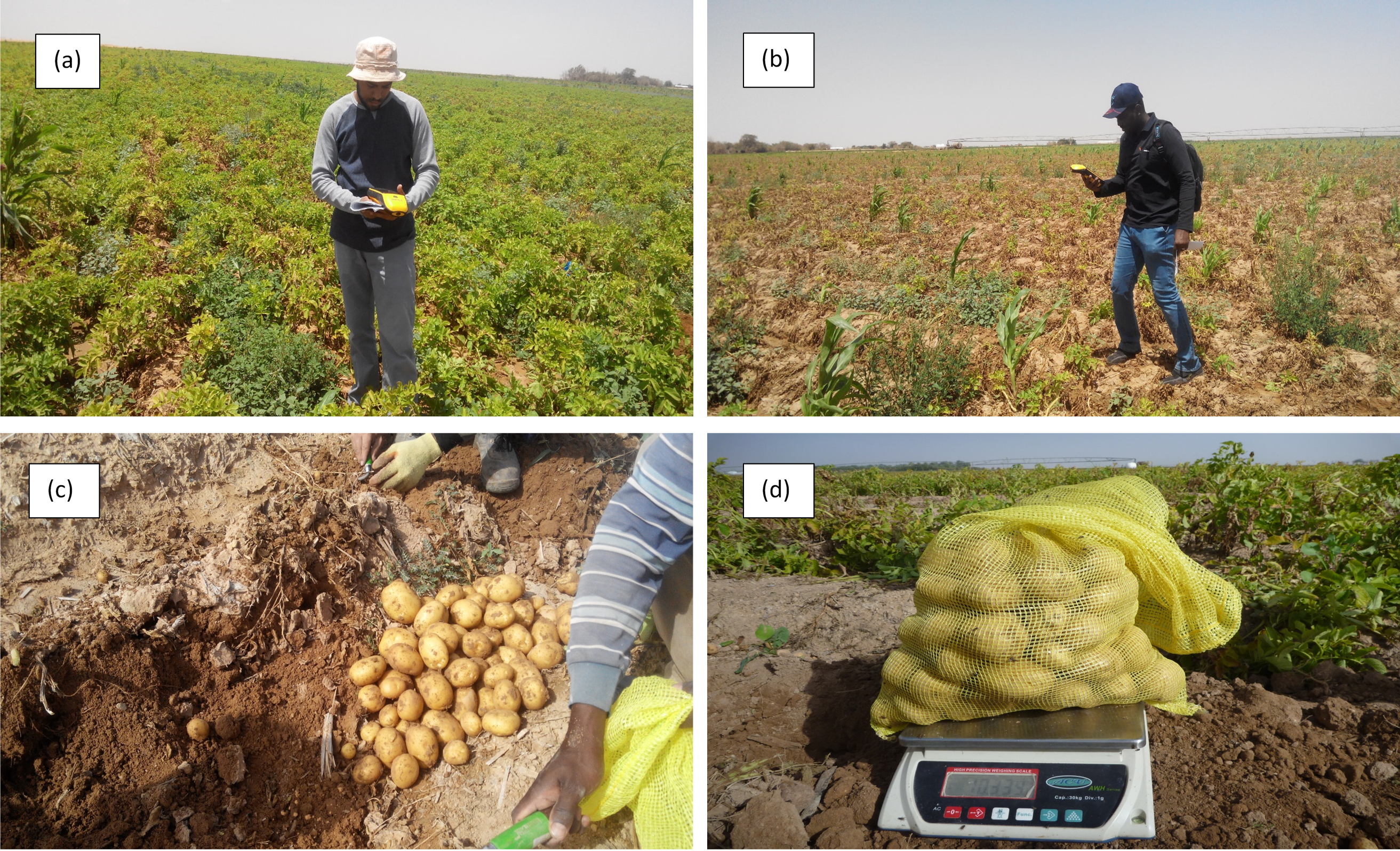

Supplement: S1 Fig — (TIF) [file pone.0162219.s001.tif]
